# Skeleton-Based Attention Mask for Pedestrian Attribute Recognition Network

**DOI:** 10.3390/jimaging7120264

**Published:** 2021-12-04

**Authors:** Sorn Sooksatra, Sitapa Rujikietgumjorn

**Affiliations:** National Electronic and Computer Technology Center, National Science and Technology Development Agency, Pathum Thani 12120, Thailand; sorn.soo@nectec.or.th

**Keywords:** pedestrian attribute recognition, pose estimation, attention network

## Abstract

This paper presents an extended model for a pedestrian attribute recognition network utilizing skeleton data as a soft attention model to extract a local feature corresponding to a specific attribute. This technique helped keep valuable information surrounding the target area and handle the variation of human posture. The attention masks were designed to focus on the partial and the whole-body regions. This research utilized an augmented layer for data augmentation inside the network to reduce over-fitting errors. Our network was evaluated in two datasets (RAP and PETA) with various backbone networks (ResNet-50, Inception V3, and Inception-ResNet V2). The experimental result shows that our network improves overall classification performance with a mean accuracy of about 2–3% in the same backbone network, especially local attributes and various human postures.

## 1. Introduction

Nowadays, image analysis of a surveillance system has gained attention in a wide range of possible aspects. Pedestrian attribute recognition (PAR) is one of the well-known areas of research that are deployed in many applications (e.g., person retrieval [1], person re-identification [2], video-based business intelligence [3], pedestrian detection [4], and so on). The research generally focuses on several attribute predictions, including personal appearances (e.g., gender, clothing, action, and so on), from a given pedestrian image. There are several challenges such as occlusions, imbalanced data distribution, camera viewpoints, low resolutions, lighting conditions, and blurred images.

**Figure 1:**
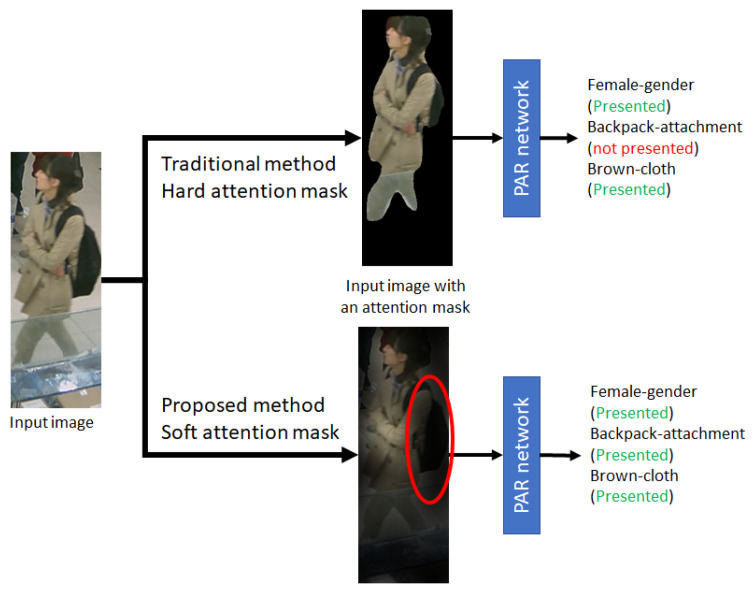
An example of pedestrian image from RAP dataset [9] for PAR network consisting of input image with hard attention mask (Mask-RCNN [7]) and with our soft attention mask.

In recent research, deep learning has dominated the PAR research. Since PAR has multiple output attributes, multi-label [5], and multi-task learning [6] are used in PAR for handling binary and multi-class classification, respectively. However, the variants of attributes in PAR effect the performance greatly. Therefore, partial image classification was included in PAR to focus the local feature of each attribute and reduce the effects of image conditions. To be specific, this idea helps to reduce the region of interest (ROI) and categorize a corresponding area to a specific attribute. Recent studies applied partial image classification in pixel-level or hard attention (human parsing) [7,8] for extracting foreground regions as shown in the top row of Figure 1. However, the region surrounding the target might contain valuable information for global attributes (e.g., gender, age, career, and so on).

This paper tackles the viewpoints and human postures in the pedestrian image by proposing an extended model for PAR. The proposed method utilized several attention masks to extract local features in each body part (e.g., head, upper body, lower body, and so on). All of the attention masks and their networks are considered as a human-part attention module, extending to the backbone network. Attention masks should be formulated to focus on a specific human body parts purpose by calculating the human skeleton confidence maps. Since skeleton data were applied, the local feature can be extracted to the human part, and it helped to handle occlusion circumstances. Skeleton joint locations can be estimated from the pedestrian image with partial occlusion. In addition, PAR could be less sensitive to posture because skeleton data can handle a large variety of human pose.

With the proposed soft attention mask, the attachment-attribute (e.g., backpack, hat, and so on) are visualized, and its local features can be extracted, as the backpack shown within a red circle of Figure 1. In case of missing skeleton data, holistic features extracted by a backbone network help to aid the human-part attention module. Our contributions are summarized as follows:

- The proposed method presented a soft attention mask formulated by skeleton data, which is insensitive to variation in human posture.
- Besides local features from a soft attention model, features from the neighboring background regions are kept for handling various viewpoints and postures.

## 2. Related Work

### 2.1. Pedestrian Attribute Recognition

In recent years, there is considerable interest in pedestrian attribute recognition (PAR). Inspired by object or image classification, previous studies trended to utilize deep-learning-based techniques or CNN models (e.g., AlexNet, VGG, Inception, and so on). PAR usually applies to multi-task learning for classifying multiple pedestrian attributes in a single image, where each attribute was indicated as a specific task in ACN [6]. ACN proposed a method to jointly train a monolithic CNN to all attributes. DeepMAR [10] exploited the relations among pedestrian attributes effectively. Latent task matrix [11] was introduced to leverage the grouping information for encouraging attributes in the same groups and enhancing a deep CNN structure allowing different CNN models to share knowledge through multi-task learning.

Besides simple deep learning-based techniques for a single image, the part-based method classified attributes in decomposed regions from the pedestrian image (i.e., hat-wearing and pant style are expected to appear at specific regions). Part-based models used object detection to decompose a human image before feeding it into the PAR network. PANDA augmented deep convolutional networks to have input layers based on semantically aligned part patches, where attributes were classified in decomposed regions from the whole image. Multi-label learning was concerned in MLCNN [12] to match classification results in multiple CNNs from each attribute. The image was decomposed by object detection to classify the body part in AAWP [13]. To avoid object detection from a preprocessing step, ARAP [14] proposed an end-to-end learning approach for a local feature in attribute recognition. Pose and background information in decomposed regions were taken into account by PGDM [15] and DHC [16]. The combination of local and global features from decomposed and whole regions was analyzed in LGNet [17]. The co-attentive sharing module introduced in [18] help to extract discriminative channels and spatial regions for more effective feature sharing for each task. The time complexity was also concerned with utilizing DS-CNN [19] to reduce the number of model parameters of PAR network.

### 2.2. Visual Attention Model

Since the complex visual scene might hardly localize the valuable features, visual attention models were added to remove the background’s interference and found the most discriminative feature within the pedestrian image. Unlike the part-based method, the attention module was generally implemented at multiple levels of the classification network formulating the attention mask as a region of interest. The well-known attention model is Faster R-CNN [20] for localization and is applied in simple CNN attention mechanisms used as units in CNN model to reduce over-fitting error. The attention model were applied on convolutional feature maps on both channel-wise [21] and spatial [22] forms.

In terms of PAR, the visual attention model was firstly introduced in HydraPlus-Net [23] for training multi-level and multi-scale features to handle various camera viewpoints and image resolutions. To take full advantage of the attention mechanism, the attention module was applied to different model levels, where their model fused several features from relevant regions and yield attention maps. The class activation map (CAM) is an important part of PAR proved by the CAM network [24]. CAM could be refined and exploited for attribute classification. Multiple attention maps [25] were assigned in different aspects, including human parsing, attribute label, and global perspective. These three attention maps combined in parallel showed the most promising performance proven by the experiment [25]. Recurrent neural network (RNN) was applied in [26] to learn context correlations and attention model capability. Feature pyramid network was utilized to solve the problem where attributes are distributed in different locations in feature pyramid attention model [27]. VESPA [28] and VALA [29] utilized a view predictor to categorize the view information (e.g., front, back, and side views). Then, each view has its classificaiton network.

### 2.3. Human Skeleton and Pose Estimation

With recent works on human and pose detection, spatial and motion features might be insufficient for handling various human postures. Recent development in human or pose detection has led to skeleton data by focusing the location and movement of human joints. This research was started in DeepPose [30] utilizing a cascade of CNN for human detector and human joint estimation. In OpenPose [31], part affinity fields (PAFs) were included for learning associate body parts in each pedestrian images. The recent approach via bottom-up method was proposed in OpenPifPaf [32] by also adding Part Intensity Field (PIF) to localize and associate human body parts, respectively. This scheme is able to store fine-grained information on low-resolution activation maps.

## 3. Attention Mask

**Figure 2:**
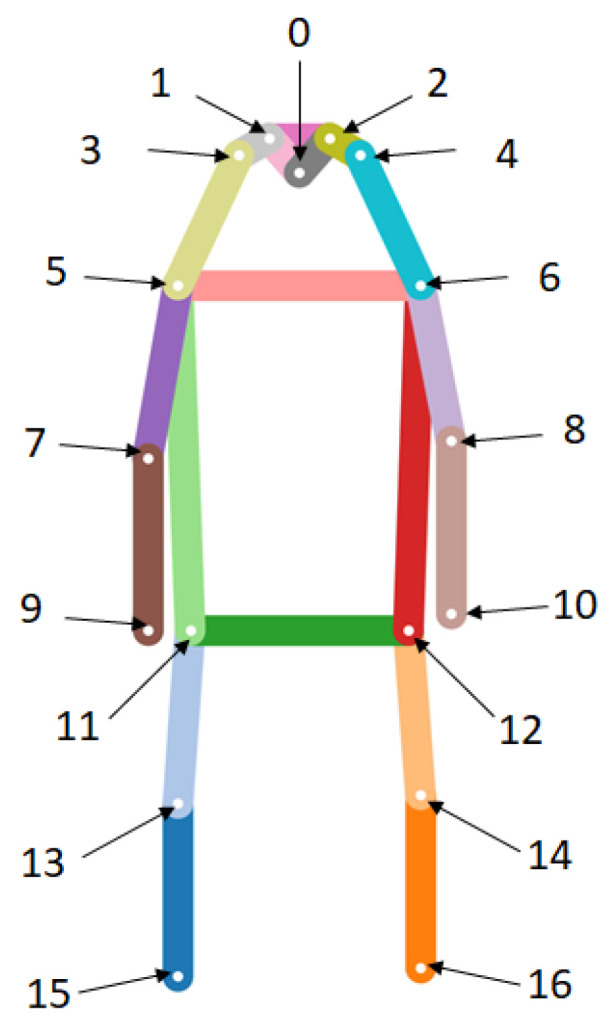
The list of skeleton names and indices from OpenPifPaf [32] with their locations.

In this section, we focused on designing an attention mask from the skeleton information to reduce the region of interest for each attribute. Our human skeleton was constructed based on OpenPifPaf [32] which had a promising human-joint localization performance. In this paper, the attention mask was extracted from their pretrained OpenPifPaf network as a separate module from PAR network. The skeleton joints were extracted and utilized for generating attention masks, where there were 17 joints as shown in Figure 2 and each joint order are as follows:
0. Nose5. Left shoulder10. Right wrist15. Left ankle1. Left eye6. Right shoulder11. Left hip16. Right ankle2. Right eye7. Left elbow12. Right hip
3. Left ear8. Right elbow13. Left knee
4. Right ear9. Left wrist14. Right knee


Based on RAP dataset [9], the attention mask was categorized into four classes (head, upper body (UB), lower body (LB), and foot) as summarized in Table 1.

**Figure 3:**
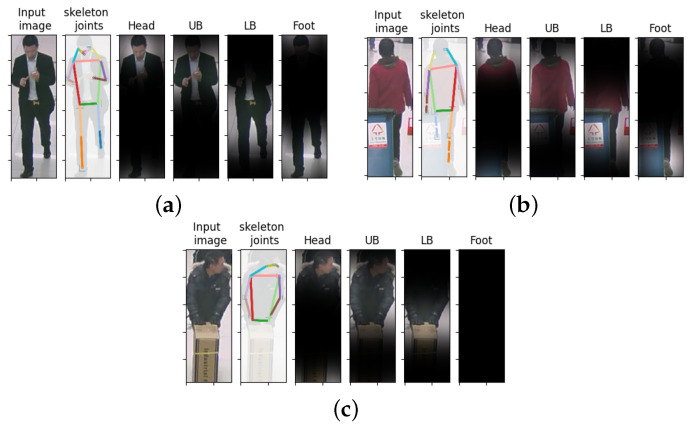
The pedestrian image with soft attention masks corresponding to attention masks in different classes where (**a**) all joints can be localized, (**b**) some joints can be detected from the partial occlusion, and (**c**) there is full occlusion.

Equation (1) calculates an attention mask with class index ‘*c*’ ($A_{c}$), where $L_{j}$ is the skeleton joint with index ‘*j*’, G(σ) is 2D Gaussian kernels with standard deviation (‘σ’), and $N_{c}$ is the number of skeleton joint in each class, resulting in the average values of the convolution between skeleton joints and Gaussian distribution. Then, attention masks would be multiplied with feature maps to indicate ROI. The examples of input images, its skeleton joint, and ROI from each class are illustrated in Figure 3a. With OpenPifPaf, the hidden joints in partial occlusion can be estimated from their visible neighbor joints as dash lines (joint indices 13 and 16) in Figure 3b. However, we decided to ignore the attention mask of that part if the full occlusion occurred at the specific area as shown in Figure 3c. The resolutions of these attention masks also needed to be adjusted for the next step to match the size of input feature. Since the attention masks have the same resolution, σ is fixed as 30 in the experiment, which is a suitable value for generating masks, especially in an upper-body region.

(1) $$A_{c}=\left(\frac{1}{N_{c}}\right)\sum_{j\in c}SIGMOID\left(L_{j}*G\left(\sigma \right)\right)$$

## 4. PAR Network Architecture

**Figure 4:**
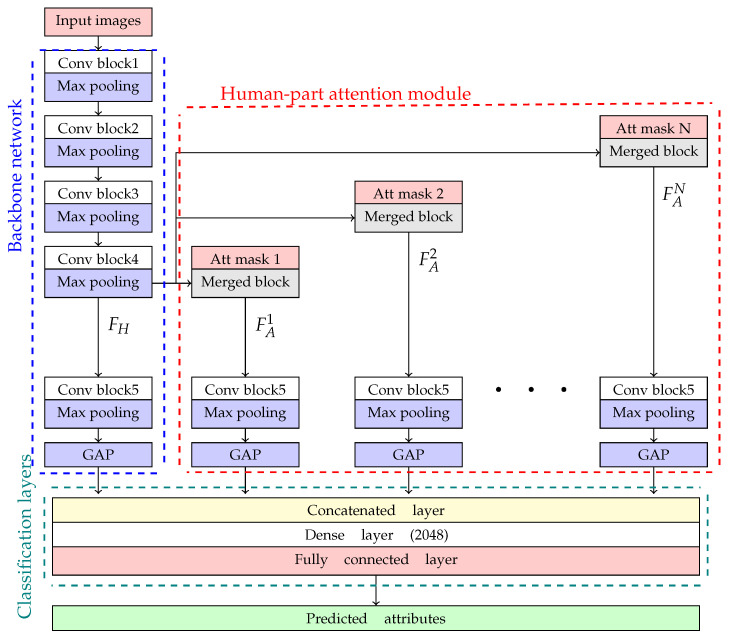
The proposed network architecture with a human-part attention module.

The overall proposed network architecture for PAR is shown in Figure 4, where networks fed by attention masks are the proposed approach in this paper. The proposed framework can be categorized into three parts consisting of the backbone network, human-part attention module, and classification layer. The first part was a baseline network utilizing well-known CNN focusing on global attributes from pedestrian images. The second part helped to extract local features in specific regions corresponding to attention masks. In the last part, classification layers received output features from the backbone network and human-part attention module to evaluate and predict attributes as the final output. The details for each part are described as follows:

### 4.1. Backbone Network

In this paper, the backbone network was constructed based on ResNet-50 [33], Inception v3 [34], and Inception-ResNet [35], which were the baseline network for PAR [36], shown within a blue rectangular box in Figure 4. The last pooling layers of the proposed network were replaced with global average pooling (GAP). The backbone network consisted of several Conv blocks, which were a sequence of Conv layers from an original study. To insert attention masks generated from the previous section, the backbone network was divided into head and tail networks. The head network consists of Conv block 1 to Conv block 4. The tail network consists of Conv block 5 to GAP.

Firstly, the input image was fed into head networks to extract low-level features from the whole image. Its output feature maps ($F_{H}$) were fed into a tail network to extract high-level features. Then, these features were fed into GAP and combined with output features from a human-part attention module (Section 4.2) resulting in the holistic feature $F_{L}^{0}$.

### 4.2. Human-Part Attention Module

As shown in Figure 4, the human-part attention module is an extended network to the backbone network. This module was applied to extract local features, which was $F_{H}$ with ith soft attention mask (Att mask *i*). The input data of this module were the multiplication between attention masks and output feature maps from a head network. The local features were extracted by tail networks within a red rectangular box in Figure 4, where they had the same number of trainable parameters. Similar to the backbone network, the feature maps from tail networks were fed into GAP as the final results in this module as $F_{A}^{i}$ corresponding to Att mask *i*.

**Figure 5:**
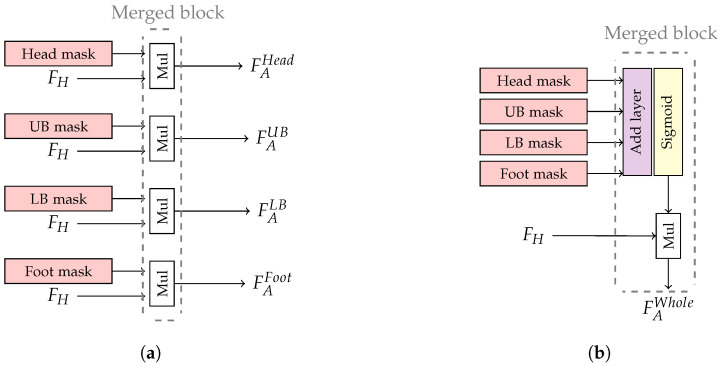
The illustration of human-part attention module for (**a**) separated and (**b**) single masks.

In the experiment, the performance of this module was expected to handle the variation of human poses from specific human parts. The human-part attention module was implemented into two versions consisting of separated and single masks. The separated mask utilized four attention masks consisting of Head, UB, LB, and Foot masks for extracting specific local features related to head, upper body, lower body, and foot attributes, respectively, where the merged blocks were represented as multiplication layers as shown in Figure 5a. The local feature in each body part can be optimized independently. On the other hand, the single mask combined all four attention masks to capture features within their overlapping regions or share local feature from different body parts as shown in Figure 5b. Moreover, an activation layer (‘Sigmoid’) was inserted to normalize the attention masks.

### 4.3. Classification Layers

After extracting visual features from the backbone network and human-part attention module, the classification layers (within a green rectangular box in Figure 4) decoded these features into predicted human attributes as the final result. The output feature maps from tail networks were fed into classification layers by a concatenated layer. With this technique, the output features from a backbone network could be fulfilled when the skeleton data could not be found in some cases. In the last layer, dense layers received the combined features and reduced the number of trainable parameters into 2048. The output from the FC layer was then sent to an activation layer (‘Linear’) for deciding whether they were categorized into positive (presented) or negative results (not presented).

## 5. Training Method

This section describes the detail of training process and model parameter optimization. Our training method aims to deal with the issue of unbalanced data of human attributes as following:

### 5.1. Network Optimization

As mentioned in Section 4.3, the final layer after the FC layer utilized ‘Linear’ as an activation layer. Since the range of the output was (−∞, *∞*), the stable binary cross-entropy (SBCE) loss function [37] was used in this paper, where negative and positive outputs were represented as non-present and present attributes, respectively. The loss function (*L*) was formulated in Equation (2):

(2) $$L\left(\hat{y},y\right)=\sum_{i=0}^{N}\omega_{i}\left(max\left({\hat{y}}_{i},0\right)-\left({\hat{y}}_{i}\right)\left(y_{i}\right)+log\left(1-e^{|{\hat{y}}_{i}|}\right)\right)$$

where (${\hat{y}}_{i},y_{i}$) is predicted and actual results from the ith attribute, respectively, and $y_{i}$ is a binary value either 0 or 1. With the unbalanced data, they cause over-fitting errors for attribute classification. Therefore, Equation (2) includes positive weights ($\omega_{i}$) to reduce the effect of attributes with several negative samples. $\omega_{i}$ is calculated in Equation (3), where $r_{i}$ is a positive ratio of the ith attribute.

(3) $$\omega_{i}=\left\{\begin{array}{cc} e^{1-r_{i}}, & \mathrm{if}\;y_{i}=1\\ e^{r_{i}}, & \mathrm{Otherwise} \end{array}\right.$$

**Figure 6:**
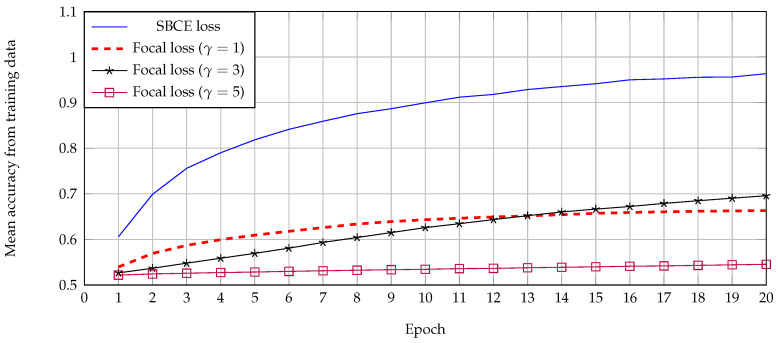
Mean accuracy from training data by using stable binary cross-entropy and focal losses with various values of γ.

As far as we know, focal loss [38] is a loss function designed for balancing between easy and hard examples from positive and negative samples in object detection. Unlike SBCE, the focal only supported the binary output which had a range [0, 1] from a PAR network utilizing ‘Sigmoid’ as an activation layer. To show the effect of the stable binary cross-entropy loss function, their mean accuracy from training data was compared with focal loss in various configuration, as shown in Figure 6. This simulation result showed that SBCE was able to achieve a higher accuracy at a lower epoch. This effect might be caused by the range of PAR network, where (−∞, *∞*) has a wider range for reducing the attribute classification error in the earlier stage.

### 5.2. Human Attribute Augmentation

Besides network optimization, the variation of training data can help reducing the effect of over-fitting errors. With unbalanced data, the number of training data from some attributes might be insufficient because of a low positive sample ratio. The training data could be augmented by their data modification using image processing techniques (e.g., flipping, blurring, rotating, and so on). The augmentation can be performed offline to generate a larger dataset which also requires a larger storage for those augmented images. On the other hand, augmentation can be performed on-the-fly during training which is also more dynamics.

**Figure 7:**
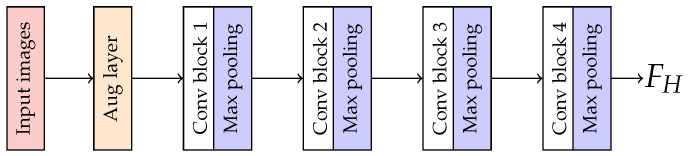
The illustration of an augmented layer in the proposed network architecture.

With this issue, we use on-the-fly augmentation by inserting an augmented layer into PAR network, between an input layer and Conv block 1 while training, as shown in Figure 7. This augmented layer was utilized for modifying and transforming incoming training data randomly with image processing techniques. Therefore, this technique helps to increase the variation of the training data while keeping the number of training data.

**Figure 8:**
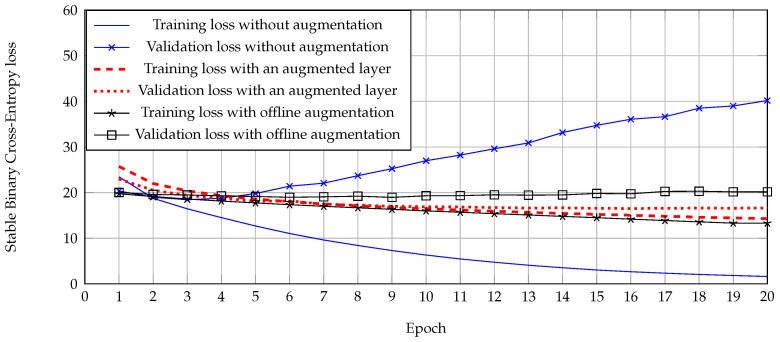
Learning curve between stable binary cross-entropy loss and epoch while training the network from RAPv2.

To show the effect of augmented layers, the learning curve between training and validation samples was analyzed as shown in Figure 8. Offline augmentation was also included as a traditional augmentation. This graph shows that the network optimization without data argumentation has a very high validation loss compared to its training loss. It caused an over-fitting error or a low classification performance in the testing samples. On the other hand, the difference between training and validation loss was very reduced by using an augmented layer compared with traditional augmentation. Therefore, data augmentation by these techniques can be practically used to reduce the effect of over-fitting errors and be more suitable in the issue.

## 6. Experiment

### 6.1. Dataset

In the experiment, our proposed network was evaluated on the two large public pedestrian datasets consisting of PETA [39] and RAP datasets [9]. The first dataset contained 19,000 pedestrian images collected from 10 small-scale person datasets which were used for person re-identification. Their image resolutions were between 17×39 and 169×365. Those images included 8705 persons, each annotated with 61 binary and 4 multi-class attributes. Training, validation, and testing samples were randomly partitioned into 9500, 1900, and 7600 images, respectively. The second dataset contained 84,928 pedestrian images and 2589 person identities with resolution ranging from 31×81 to 415×583. There were 54 selected attributes for evaluation. The images were captured from surveillance cameras with high definition (1280×720) and 25 camera viewpoints. All samples were categorized into 50,957, 16,986, and 16,985 images for training, validation, and testing, respectively. Their attributes from PETA and RAP dataset were categorized as shown in Table 2 and Table 3, respectively.

### 6.2. Implementation Detail

In preprocessing step, the skeleton data were first formulated from pedestrian images by utilizing the pretrained OpenPifPaf network. Then, attention masks were calculated by Equation (1) and resized into 16×16. The pedestrian image was resized to 250×250 before feeding into the PAR network. In network optimization, the PAR network was optimized by Equation (2) and utilized three backbone networks consisting of ResNet-50, Inception V3, and Inception-ResNet V2 (I-ResNet V2) which were well known in PAR research for evaluation. The training method was operated in 20 epochs by declining a learning rate from $1\times 10^{-2}$ to $1\times 10^{-4}$ with weight decay = $5\times 10^{-4}$. To solve the problem of identical identities among training, validation, and testing data, zero setting [37] was utilized to repartition images from PETA and RAPv2 datasets.

### 6.3. Evaluation Metric

The evaluation metric used for PAR consisted of Recall, Precision, F1, and mean accuracy (mA), calculated in Equations (4)–(7), respectively.

(4) $$\mathrm{Recall}=\frac{TP}{TP+FN}$$

(5) $$\mathrm{Precision}=\frac{TP}{TP+FP}$$

(6) $$F1=2\times \frac{\mathrm{Recall}\times \mathrm{Precision}}{\mathrm{Recall}+\mathrm{Precision}}$$

(7) $$\mathrm{mA}=\frac{TP+TN}{TP+FN+FP+TN}$$

where TP, FN, FP, and TN are the number of true positives, false negatives, false positives, and true negatives, respectively. From the above equations, F1 was utilized for evaluating the classification of positive samples. On the other hand, mA was used for both positive and negative samples, where mA also analyzed the effect of unbalanced data.

### 6.4. Experimental Results

#### 6.4.1. Overall Performance

As mentioned in the introduction, this paper focused on an extension module to improve the attribute classification performance for the PAR network. In the experiment, the backbone network consisting of ResNet-50, Inception V3, and I-ResNet V2 were used with various configurations as shown in Table 4 and Table 5 for RAP and PETA datasets, respectively.

By applying only the backbone network, Table 4 and Table 5 showed that ResNet-50 had better classification performance than inception V3 and I-ResNet V2. For networks with attention masks, the single mask outperformed other configurations, especially in Recall and mA. It indicated that more true positive samples were presented in this configuration. In the PETA dataset, I-ResNet V2 with single mask had a significant improvement from its backbone network, while its result was ineffective in RAP dataset. This issue might be caused by its inappropriate configuration or high diversity in the RAP dataset. On the other hand, the separated mask is less effective on both datasets, especially on Inception V3 and I-ResNet V2. The problem might be from their large number of parameters with separated mask compared with other configurations, resulting in higher over-fitting errors.

#### 6.4.2. Attribute-Level Performance

Since the PAR network was designed as a multi-task learning network for handling several predicted attributes or outputs. The overall performance might be insufficient for PAR evaluation. The classification performance should be visualized in the attribute level to show the effectiveness of global and other local attributes. Since mA was used for both classification in negative and positive samples, this evaluation was relied on mA as shown in Table 6 and Table 7 for RAP and PETA dataset, respectively. Similar to the overall performance mentioned above, the PAR network with a single mask outperformed other configurations in most categorizations. However, I-ResNet V2 with and without attention masks had insignificant differences in local attributes from the RAP dataset. Comparing the backbone network, ResNet-50 performs slightly better than other two networks in both datasets.

The experimental results showed that global attributes were less effective than other local attributes. The significant improvement from the proposed method is attributes located on a large region of the body, upper and lower bodies. In some viewpoints, especially on the back of a person, the attributes may not be clearly visible; therefore, their salient features from the facial area could not be extracted for classifying their attributes (e.g., gender, age, glasses, and so on). In addition, the attributes related to action have the worst performance in RAP dataset. It indicated that the motion information should be taken into account for this matter. Even though some attributes related to attachment (e.g., backpack, plastic bags, and so on) might not be located within human bodies, the performance from the single mask was slightly improved, where their features could be extracted from the soft attention mask while ignoring the further background region.

#### 6.4.3. Time Complexity

This section described the time consumption for the PAR network with and without attention masks. In the experiment, our hardware specification was Intel(R) Xeon(R) Gold 6148 CPU @ 2.40 GHz with 2x Nvidia Tesla V100 for PCIe. Table 8 shows the frame rate or frame per second (fps) for PAR networks run in this experiment. It shows that the time complexity is directly proportional to the size of PAR network, where ResNet-50 has the highest frame rate among three networks. With attention masks, it reduces the frame rate by about 5% and 6% for single and separated masks, respectively. Even though all frame rate satisfied the minimum requirement for real-time video (25 fps), these networks should be operated in on-field hardware specification to ensure practical usage.

## 7. Discussions

**Figure 9:**
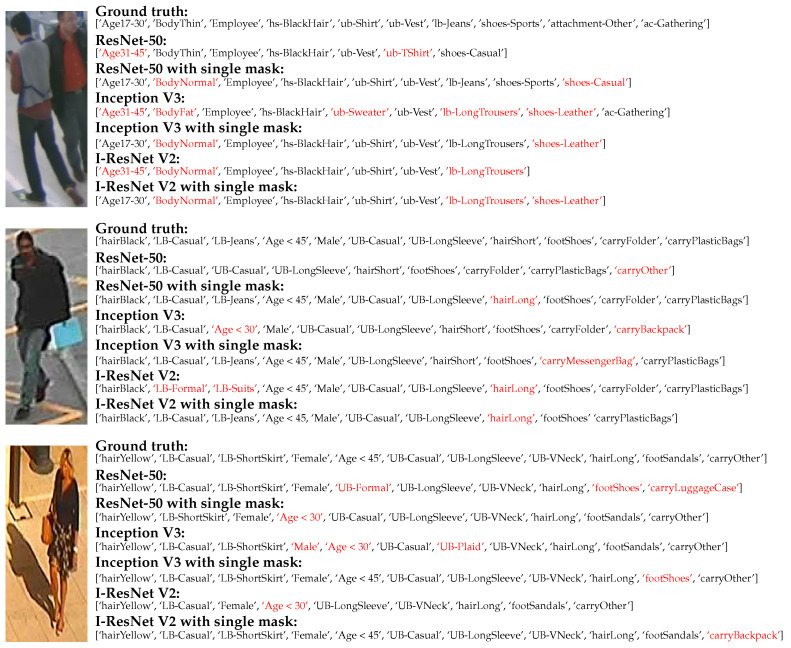
The examples of the predicted attribute on pedestrian images with normal circumstance with various race, where the wrong attributes are presented as red characters.

This section analyzed the predicted result from PAR networks and factors of attribute classification performance. The PAR network with and without attention masks were evaluated to show their merit and demerit. Figure 9 shows an example of predicted attributes from pedestrian images in the normal circumstance (standing pose without occlusion). They shows that most predicted attributes were presented from people with different race and gender in RAP and PETA dataset. However, there are specific conditions affecting our attention masks and the attribute classification, which can be summarized as follows.

### 7.1. Surrounding Region

Since the role of the attention mask is to extract salient features within interested regions, our method is expected to ignore background or surrounding regions in the experiment. Not only was the surrounding object discarded, false targets should be discarded as well. As you can be seen in Figure 9 (the first row), more attributes were presented by the backbone network with a single mask. Since our target was selected from the size of the skeleton mask, the attributes of an actual target (the man on the left) can be presented, especially on the age attribute. On the other hand, the PAR networks without attention masks might obtain another target feature (the man on the right) because the wrong age was presented.

### 7.2. Occlusion

**Figure 10:**
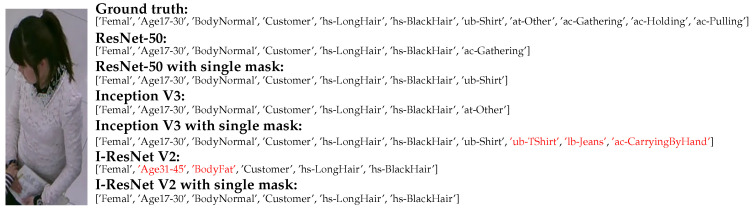

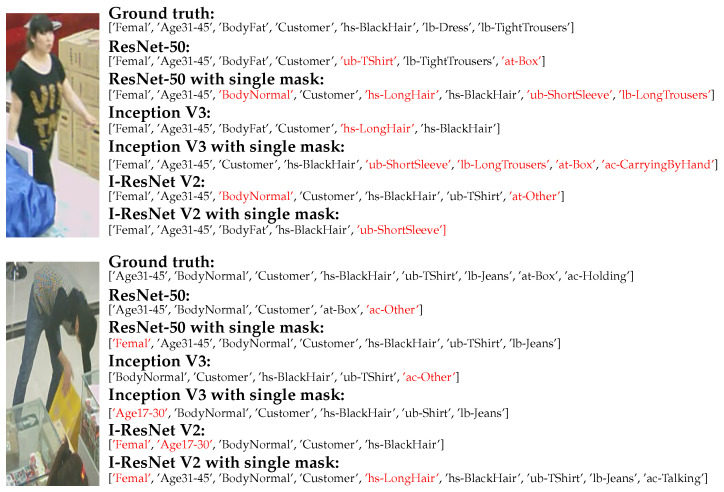
The examples of predicted attribute from pedestrian images corresponding to full occlusion, partial occlusion, and irregular posture in the first, second, and third rows, respectively, where the wrong attributes are presented as red characters.

The occlusion is another problem in PAR research where it is categorized as fully and partial occlusion. According to the RAP and PETA dataset ground truths, the attributes which are fully occluded within the image expected to be removed in the predicted result. Figure 10 (the first row) shows most predicted results from the proposed method are able to ignore attributes from lower-half body which is fully occluded. For Inception V3 with single mask, their wrong predicted attributes might be caused by the carried objects in the image from soft attention mask. On the other hand, the visual feature from partial occluded objects (e.g., table, box, and so on) might reduce the classification performance on the PAR network. With attention masks, those features cannot be ignored resulting in the correct predicted attributes. Figure 10 (the second row) shows the results of the pedestrian image with a partial occlusion on the lower body, but the attribute related to the lower body can be presented by our method.

### 7.3. Irregular Human Posture

Another merit from skeleton data is the robustness to human posture. Therefore, it is expected that our method could be insensitive to any human motion or posture in the pedestrian image. As far as we observed, most of the human posture is standing and walking as regular postures in the dataset. It causes the unbalanced distribution to other or irregular postures (e.g., bending down, sitting, and so on). The visual feature in some attributes might not be localized in the pedestrian image with irregular postures. Figure 10 (the third row) is the example for this matter as a bending down posture, where the backbone network might not localize visual features in the attribute related to the lower body. On the other hand, our networks were able to localize the lower body position, where the actual attribute (‘lb-Jeans’) can be presented. However, global attributes (e.g., age, gender, and so on) are effected due to smaller ROI from soft attention mask.

## 8. Conclusions

This paper described the extended module for the PAR network with a soft attention module. A human-part attention module was implemented, where it consisted of several tail networks corresponding to the human body part. The attention mask was formulated by skeleton data to capture local features from intermediate Conv layers from a head network and to handle various video conditions, especially in the human posture. In addition, the augmented layers as data augmentation were included to randomize the image condition of feeding data inside the PAR network and reduce the effect of over-fitting error. The proposed network was evaluated on two datasets (RAP and PETA) with three backbone networks consisting of ResNet-50, Inception V3, and Inception-ResNet V2. The empirical results showed that the proposed method outperformed their backbone networks, especially with a single mask in Recall and mA. In the predicted attribute analysis, it showed that our method could extract more valuable information than the baseline methods without attention masks, on the large region (upper and lower body) and was insensitive to human postures in local attribute prediction.

Even though our overall performance outperforms the results from the method with the same backbone network, some specific attributes, especially in human action, could not achieve the promising performance, especially in Inception-ResNet V2 which has a large number parameter causing over-fit errors. In future work, we plan to implement an attention mask inside the PAR network to generalize our network for practical applications and to improve classification performance, especially in global attributes.

## Figures and Tables

**Table 1:** The class of attention mask corresponding to skeleton join index.

Class Index	Class Name	List of Skeleton Joint Index
1	Head	0, 1, 2, 3, 4
2	Upper body	5, 6, 7, 8, 9, 10
3	Lower body	11, 12, 13, 14
4	Foot	15, 16

**Table 2:** Attribute categorization on PETA dataset.

Group	Attribute
Global (G)	gender, age
Head (H)	hair length, muffler, hat, glasses
Upper body (U)	clothes style, logo, casual or formal
Lower body (L)	clothes style, logo, casual or formal
Foot (F)	footware style
Attachment (At)	backpack, messenger bag, plastic bags.

**Table 3:** Attribute categorization on RAP dataset.

Group	Attribute
Global (G)	gender, age, body shape, role
Head (H)	hair style, hair color, hat, glasses
Upper body (U)	clothes style, clothes color
Lower body (L)	clothes style, clothes color
Foot (F)	footware style, footware color
Attachment (At)	backpack, single shoulder bag, handbag
Action (Ac)	telephoning, gathering, talking, pushing

**Table 4:** Ablation study for the proposed method on RAP dataset.

Networks	Evaluation Metric			
	Recall (%)	Precision (%)	F1-Score (%)	mA (%)
ResNet-50	50.88%	61.77%	55.80%	71.86%
ResNet-50 with single mask	**55.83%**	**62.12%**	**58.81%**	**74.19%**
ResNet-50 with separated mask	45.05%	54.71%	49.41%	73.33%
Inception V3	49.82%	59.72%	53.95%	71.21%
Inception V3 with single mask	**54.06%**	**60.47%**	**57.08%**	**73.32%**
Inception V3 with separated mask	50.04%	56.68%	53.15%	71.25%
I-ResNet V2	51.66%	55.41%	54.45%	72.00%
I-ResNet V2 with single mask	**53.16%**	59.00%	**55.93%**	**72.17%**
I-ResNet V2 with separated mask	51.23%	**59.90%**	53.47%	72.08%

**Table 5:** Ablation study for the proposed method on PETA dataset.

Networks	Evaluation Metric			
	Recall (%)	Precision (%)	F1-Score (%)	mA (%)
ResNet-50	56.09%	69.20%	61.96%	76.33%
ResNet-50 with single mask	**58.02%**	**70.80%**	**63.77%**	**77.16%**
ResNet-50 with separated mask	55.43%	62.92%	58.94%	76.90%
Inception V3	54.05%	**68.85%**	60.56%	75.26%
Inception V3 with single mask	**56.76%**	65.86%	**60.97%**	**76.44%**
Inception V3 with separated mask	53.15%	65.12%	58.53%	74.46%
I-ResNet V2	51.55%	62.27%	54.77%	73.51%
I-ResNet V2 with single mask	**55.77%**	**66.56%**	**59.73%**	**76.09%**
I-ResNet V2 with separated mask	49.34%	63.43%	53.43%	72.40%

**Table 6:** The attribute classification performance in mA from RAP dataset.

	Global	Head	Upperbody	Lowerbody	Foot	Attachment	Action
ResNet-50	68.46%	80.44%	73.10%	77.88%	70.93%	71.60%	66.32%
ResNet-50 with single mask	**71.12%**	**80.52%**	**75.89%**	**82.49%**	**73.52%**	**74.05%**	**67.22%**
ResNet-50 with separated mask	66.65%	72.14%	70.90%	81.19%	71.17%	63.70%	60.95%
Inception V3	68.61%	77.14%	72.43%	79.90%	68.74%	70.25%	66.15%
Inception V3 with single mask	**70.98%**	**77.63%**	**75.40%**	82.28%	**71.55%**	**73.14%**	**66.57%**
Inception V3 with separated mask	69.81%	75.61%	73.96%	**83.78**%	70.73%	66.70%	63.43%
I-ResNet V2	68.43%	**77.99%**	73.60%	**79.76%**	68.21%	72.66%	**67.62%**
I-ResNet V2 with single mask	**71.66%**	75.83%	**74.50%**	78.59%	68.37%	72.40%	66.17%
I-ResNet V2 with separated mask	68.59%	75.31%	73.07%	82.13%	**70.80%**	**72.82%**	66.53%

**Table 7:** The attribute classification performance in mA from PETA dataset.

	Global	Head	Upperbody	Lowerbody	Foot	Attachment
ResNet-50	74.68%	69.89%	**80.76**%	78.36%	71.23%	78.57%
ResNet-50 with single mask	**75.25%**	**72.29%**	80.47%	**78.94%**	**72.84%**	**79.39%**
ResNet-50 with separated mask	74.53%	68.96%	79.17%	78.64%	70.97%	77.45%
Inception V3	74.76%	68.79%	79.54%	77.73%	69.73%	77.66%
Inception V3 with single mask	**74.81%**	**69.42%**	**79.86%**	**79.74%**	**71.48%**	**78.74%**
Inception V3 with separated mask	74.13%	67.67%	78.53%	76.38%	69.69%	77.36%
I-ResNet V2	73.12%	67.12%	77.83%	75.11%	68.96%	75.99%
I-ResNet V2 with single mask	**74.80%**	**69.49%**	**80.65%**	**78.97%**	**70.12%**	**78.14%**
I-ResNet V2 with separated mask	73.23%	66.20%	76.34%	73.98%	68.05%	75.00%

**Table 8:** The frame rate of PAR network with and without attention masks.

	without Mask (fps)	with Single Mask (fps)	with Separated Mask (fps)
ResNet-50	38.85	37.26	36.49
Inception V3	36.49	35.17	34.80
I-ResNet V2	35.75	33.89	33.43

## Data Availability

The RAP dataset is openly available at http://www.rapdataset.com/, reference number [9] and PETA dataset is openly available at http://mmlab.ie.cuhk.edu.hk/projects/PETA.html, reference number [39].
